# Orthostatic intolerance in chronic fatigue syndrome

**DOI:** 10.1186/s12967-019-1935-y

**Published:** 2019-06-03

**Authors:** Richard Garner, James N. Baraniuk

**Affiliations:** Division of Rheumatology, Immunology and Allergy, Department of Medicine, Georgetown University Medical Center, 3900 Reservoir Rd NW Preclinical Science LD03, Washington, DC 20007 USA

**Keywords:** Chronic fatigue syndrome, Orthostatic intolerance, POTS, Tachycardia

## Abstract

**Background:**

Orthostatic intolerance (OI) is a significant problem for those with chronic fatigue syndrome (CFS). We aimed to characterize orthostatic intolerance in CFS and to study the effects of exercise on OI.

**Methods:**

CFS (n = 39) and control (n = 25) subjects had recumbent and standing symptoms assessed using the 20-point, anchored, ordinal Gracely Box Scale before and after submaximal exercise. The change in heart rate (ΔHR ≥ 30 bpm) identified Postural Orthostatic Tachycardia Syndrome (POTS) before and after exercise, and the transient, exercise-induced postural tachycardia Stress Test Activated Reversible Tachycardia (START) phenotype only after exercise.

**Results:**

Dizziness and lightheadedness were found in 41% of recumbent CFS subjects and in 72% of standing CFS subjects. Orthostatic tachycardia did not account for OI symptoms in CFS. ROC analysis with a threshold ≥ 2/20 on the Gracely Box Scale stratified CFS subjects into three groups: No OI (symptoms < 2), Postural OI (only standing symptoms ≥ 2), and Persistent OI (recumbent and standing symptoms ≥ 2).

**Conclusions:**

Dizziness and Lightheadedness symptoms while recumbent are an underreported finding in CFS and should be measured when doing a clinical evaluation to diagnose orthostatic intolerance. POTS was found in 6 and START was found in 10 CFS subjects. Persistent OI had symptoms while recumbent and standing, highest symptom severity, and lability in symptoms after exercise.

*Trial registration* The trial was registered at the following: https://clinicaltrials.gov/ct2/show/NCT03567811

## Background

Chronic fatigue syndrome (CFS) is characterized by disabling fatigue, cognitive, nociceptive, and somatosensory complaints. An important defining symptom is post-exertional malaise (PEM) with marked worsening of physical and cognitive symptoms after exertion [1–3]. The Institute of Medicine (IOM) estimates that 836,000 to 2.5 million Americans have CFS. Recently, the IOM recommended that CFS be renamed Systemic Exertional Intolerance Disease (SEID) and proposed new diagnostic criteria by requiring: (1) unexplained fatigue leading to a disability and lasting more than 6 months, (2) PEM, (3) unrefreshing sleep, (4) plus one of either cognitive impairment or orthostatic intolerance.

In SEID, orthostatic intolerance (OI) is defined by symptoms of dizziness, lightheadedness, blurred vision, and near syncope that worsen upon assuming and maintaining upright posture that are usually alleviated by recumbency. The most prevalent forms of OI are Postural Tachycardia Syndrome (POTS) and Neurally Mediated Hypotension [4]. POTS is defined as orthostatic symptoms that occur with standing and an increase in heart rate of ≥ 30 bpm (ΔHR) when moving from a recumbent position to standing, and heart rate while standing may exceed 120 bpm. Neurally Mediated Hypotension is defined as a sustained reduction of systolic blood pressure ≤ 20 mmHg or diastolic blood pressure ≤ 10 mmHg within 3 min of standing [5–7]. OI symptoms can be exacerbated by exertion and may be considered a component of post-exertional malaise. The constant postural tachycardia seen in POTS can be taxing and contribute to a lower quality of life [8, 9].

A new variant of exercise-induced postural tachycardia was found in Gulf War illness (GWI). Rayhan et al. found that one-third of GWI study participants developed transient, postural tachycardia after a submaximal bicycle stress test [10, 11]. This group was called the Stress Test Activated Reversible Tachycardia (START) phenotype. They had no postural tachycardia before exercise, and the responses disappeared 24–48 h after exercise, making it unique and different from POTS. The other two-thirds of GWI participants had no orthostatic tachycardia before or after exercise and were defined as the Stress Test Originating Phantom Perception (STOPP) phenotype. GWI shares many complaints with CFS including fatigue, cognitive dysfunction, unrefreshing sleep, PEM, pain, and orthostatic complaints [12]. The relationship between START, OI, and POTS, and occurrence in CFS are not known and was examined.

Current instruments to define OI include self-report questionnaires such as COMPASS-31, the Orthostatic Grading Scale, and visual analog scales for instantaneous evaluation of orthostatic symptoms, such as dizziness, lightheadedness, goofiness, or cognitive impairment [13, 14]. Limitations with these methods include differences in scoring based on past experiences, absent or ill-defined anchors, possibilities of ceiling and floor effects in subjects compared to controls, and measurements performed only while standing or during tilt testing [15–17]. To overcome these limitations, we adapted the standardized Descriptor Differential Scale (DDS) for pain intensity from Gracely et al. [18] that was based on psychophysical principles to provide a more structured scale to assess Dizziness and Lightheadedness. The DDS, colloquially called the Gracely Box Scale, is an anchored ordinal scale from 0 to 20 using verbal descriptors to measure symptom severity and reduce scaling error [18, 19].

The purpose of this study was to characterize OI in CFS and the effect of exercise on OI. We introduced the Gracely Box Scale as a novel, simple method to measure the Dizziness and Lightheadedness symptom severity in a clinical setting. We identified POTS and START in both controls and CFS. However, the orthostatic symptoms of Dizziness and Lightheadedness were not associated with orthostatic tachycardia in either POTS or START. We identified three subgroups of patients with orthostatic complaints in CFS, including a subgroup that reported recumbent Dizziness and Lightheadedness. Those with recumbent symptoms had higher symptom severity upon standing and greater variability in symptom severity after exercise compared to those who had symptoms only after standing.

## Methods

### Ethics statement

The protocol was approved by the Georgetown University Institutional Review Board (IRB 2013-0943), and listed in clinicaltrials.gov (NCT03567811). Sedentary control and CFS subjects were recruited between 2013 and 2016 from websites, word of mouth, fliers, newspaper and online advertisements, and personal contacts in clinics and support groups.

### Subjects

Interested sedentary control and chronic fatigue syndrome (CFS) participants responded via email or telephone (n = 183). After obtaining verbal consent, each volunteer had an initial telephone screening with a clinical research associate who read a scripted outline of the study to assess inclusion and exclusion criteria (n = 147). CFS candidates were screened for CFS using the 1994 Centers for Disease Control and Prevention criteria [1, 20]; chronic medical and psychiatric illnesses current medications. After approval, subjects were asked to complete questionnaires using our online eZhengtricity data collection system [21]. CFS diagnoses were confirmed during history and physical examination on screening day by CDC Fukuda and Canadian Consensus Criteria (n = 39) [1–3]. Non-control subjects that did not fully meet CFS criteria were further separated into Chronic Idiopathic Fatigue (CIF, n = 4) and CFS-like with insufficient fatigue syndrome (CFSLWIFS, n = 0). CIF subjects reported moderate or severe fatigue, but had ≤ 3 ancillary symptoms. CFSLWIFS reported none, trivial, or mild fatigue, but had ≥ 4 ancillary symptoms [20]. Exclusions included chronic medical illnesses that predated the onset of CFS or that could explain the full range of each individual’s symptoms, and chronic psychiatric diseases with hospitalization in the past 5 years. Subjects with any history of cardiac illness or impairment were excluded except for those with controlled hypertension. Controlled type II diabetes and thyroid disease were allowed.

### Gracely Box Scale measurements

The Descriptor Differential Scale was adapted as the Gracely Box Scale (GBS) in order to grade symptom intensity or severity (Table 1). The 0 to 20 anchored, ordinal scale was originally intended to assess pain intensity [18]. The wide range and anchors gave subjects options to grade low and high severity complaints that may have avoided floor and ceiling effects, respectively. Subjects were instructed on using the scale and choosing the optimal integer. No fractions were allowed. In this report, we focus solely on Dizziness and Lightheadedness symptoms.

**Table 1:** Gracely Box Scale

0–20	Description
20	Extremely intense
19	
18	Very intense
17	
16	Intense
15	Strong
14	Slightly intense
13	
12	Barely strong
11	Moderate
10	
9	Mild
8	
7	Very mild
6	
5	Weak
4	Very weak
3	
2	Present
1	
0	Not present

Subjects graded the severity of symptoms of Dizziness and Lightheadedness. Only integers were allowed to assess symptom severity

### Orthostatic intolerance testing

Symptoms were measured before and after orthostatic testing. Subjects reclined supine and used the GBS to scale their symptoms. Subjects remained quiet without talking for five minutes to avoid any confounding sympathetic nervous system activation. Subjects then stood up by themselves and remained ten inches from the edge of the bed for 5 min. At the end of the 5-min standing period, symptoms were graded in identical fashion. Heart rates and blood pressures were measured at 1-min intervals throughout the supine and standing phases. The identical protocol was used prior to and approximately 1, 3, 8, 16, 24, and 36 h after the first exercise stress test. Postural orthostatic intolerance was quantified as the difference between supine and standing symptoms (ΔSymptoms).

### Definitions

ΔHR was the difference between HR at each 1-min time point while standing minus the average of the 5 recumbent HR measurements. A normal ΔHR was 12 ± 2 beats per minute (mean ± SD).

ΔSymptoms was the difference between supine and standing symptoms.

Postural Tachycardia Syndrome (POTS) was defined on the screening day. POTS was defined as an increase in ΔHR ≥ 30 bpm at two or more measurements during the 5-min standing period [5–7].

START was defined by having a normal ΔHR (12 ± 2 bpm, mean ± SD) in the pre-exercise phase, but after exercise, ΔHR was increased to ≥ 30 bpm at two or more measurements during the 5-min standing periods [10, 11].

STOPP was defined by having normal ΔHR at every pre- and post-exercise time points [10, 11].

### Bicycle stress test

The Schwinn AirDyne bicycle submaximal exercise protocol was adapted from Light et al. [22]. Two tests were performed about 24 h apart. Subjects had EKG and vital signs recorded while sitting at rest for 5 min. Subjects started pedaling at a slow rate. Pedaling rate and bicycle resistance level were increased to achieve 70% of predicted maximum HR (220-age) after 3 to 5 min. The first goal was to cycle for 25 min at 70% HR. The second goal was to cycle to reach 85% predicted max HR, equivalent to a cardiac stress test, and anaerobic threshold. EKG, vital signs, and symptom scores were obtained every 5 min. Subjects stopped when they felt exhausted, reached a score of 19/20 on the Borg Exertion Scale [23], had respiratory quotient exceeding 1, or reached 85% predicted max HR. Exercise outcomes are reported elsewhere.

### Statistical analyses

Dizziness and Lightheadedness scores for CFS subjects (n = 39) were compared to a pooled control group containing CIF (n = 4) and sedentary control subjects (n = 21). There were no CFSLWIFS subjects in this study. Recumbent, standing, and ΔSymptoms were collated for each time period and plotted to assess each individual time course. Incremental increases for symptoms after exercise were measured by subtracting the pre-exercise average from each post-exercise measurement and plotting the difference against hours after Day 1 exercise. Slopes were calculated by linear regression for raw symptom scores and changes over time.

Area under the curve (AUC) for raw and incremental data was measured by the trapezoid method and normalized for each patient by dividing the value by the number of hours of recordings. Root Mean Square Error (RMSE) was measured as the square root of the mean square error of post-exercise residual values after subtracting pre-exercise values for each subject.

It is important to determine if nominal, ordinal, and smaller data sets follow a normal distribution. Nominal and ordinal symptom data was determined to not be normally distributed by Levene’s test; thus, nonparametric statistics were applied. Significant differences between groups were tested by Mann–Whitney U test. Analysis between recumbent and standing data within each group was performed using Wilcoxon Signed-Rank Test. Questionnaire data followed normal distributions and analyses between groups were performed by ANOVA followed by post hoc Tukey’s Honest Significant Difference (HSD). False Discovery Rate (FDR) was calculated to correct for multiple comparisons [24]. Significant differences for frequency data were determined by Fisher’s Exact Test (FET) and Receiver Operating Characteristics (ROC) curves. All statistical analyses were calculated using IBM^®^ SPSS^®^ Statistics 25 for Windows and Microsoft Excel. All data are reported as Mean ± SD.

## Results

### Do CFS subjects have orthostatic symptoms?

**Fig. 1:**
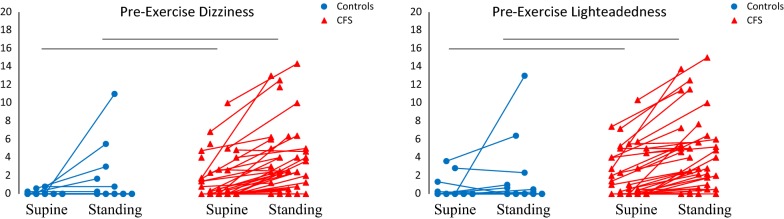
Pre-exercise Dizziness and Lightheadedness Scores. CFS subjects reported significantly more Dizziness and Lightheadedness than controls while supine and standing. Absence of symptoms was reported in 14/25 controls and 6/39 CFS subjects (FET, *p *< 0.0001). The connected dots indicate trends for Dizziness and Lightheadedness to increase in controls and CFS when standing up. Black bars indicate *p *< 0.05 by Mann–Whitney U test between CFS and controls for supine and standing symptoms

The study was completed by 39 CFS and 25 control subjects. All subjects completed questionnaires and submaximal exercise stress tests. Orthostatic complaints of Dizziness and Lightheadedness were scored while recumbent and after 5 min of standing. Dizziness and Lightheadedness were absent (scores = 0) while recumbent and standing in 56% of controls (14/25) and 15% of CFS (6/39) (FET, *p *< 0.0001, controls vs. CFS) indicating that portions of each group had no orthostatic complaints. Because the majority of control scores were 0, we initially defined a positive score as ≥ 1. Controls and CFS were compared before exercise (Fig. 1). While recumbent, Dizziness ≥ 1 and Lightheadedness ≥ 1 occurred in only 0/25 and 3/25 controls, respectively. In contrast in CFS, Recumbent Dizziness occurred in 15/39 and 18/39 subjects, respectively (FET, *p *< 0.0001, vs controls). After standing, 4/25 and 5/25 controls developed Dizziness and Lightheadedness, respectively. Again, more CFS had Dizziness (28/39) and Lightheadedness (28/39) after 5 min of standing (FET, *p *< 0.0001 CFS vs. controls). Incremental increases of 1 or more (ΔSymptoms) occurred in 4/25 controls and 25/39 CFS subjects for both Dizziness and Lightheadedness (FET, *p *< 0.0005 CFS vs. controls). Mean symptom scores were significantly greater in CFS than controls for Dizziness and Lightheadedness while recumbent, standing, and ΔSymptoms (*p *< 0.05, Mann–Whitney U test). Therefore, CFS had significant Dizziness and Lightheadedness while recumbent and standing. OI was demonstrated by the dynamic increase in Dizziness and Lightheadedness after standing up.

### Does POTS explain orthostatic complaints in CFS?

We hypothesized that CFS subjects with POTS would have higher scores after standing up than those without POTS (non-POTS). POTS was defined by postural tachycardia (ΔHR ≥ 30 bpm) plus Dizziness and Lightheadedness symptoms after standing, and was present in 1/25 controls and 6/39 CFS subjects before exercise. This rate was similar to previous findings in CFS after active standing [25, 26]. One control and one CFS subject had postural tachycardia without any OI symptoms. They represent a poorly characterized population, but were included in the POTS groups in the results below. OI symptoms without postural tachycardia while standing occurred in 3/25 controls and 21/39 CFS. Despite their symptoms, they could not be defined as POTS because of their normal ΔHR. No OI symptoms were found in 20/25 controls and 11/39 CFS while standing (*p *< 0.0005, 2 × 4 FET). More CFS subjects had OI symptoms without postural tachycardia than POTS, and the magnitudes of OI symptoms were not different between POTS and non-POTS subjects in either the CFS or control groups. Therefore, POTS did not explain the OI symptoms in the CFS cohort.

### Is orthostatic intolerance a component of Post-Exertional Malaise?

**Fig. 2:**
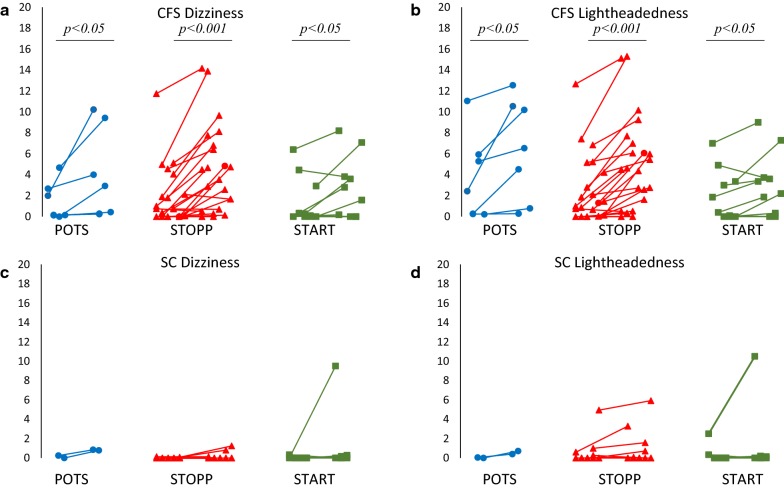
Average Recumbent and Standing Dizziness and Lightheadedness Scores in POTS, STOPP, and START. Each pair of symbols shows the average scores while recumbent (left) and standing (right). CFS POTS (blue), STOPP (red), and START (green) had significantly higher symptoms between recumbent Dizziness and Lightheadedness after 5 min of standing. In contrast, controls had no significant changes in symptoms between the average of all recumbent and standing measurements even if they had postural tachycardia before and after (POTS, blue) or only after exercise (START, green). Black bars indicate *P *< 0.05, Wilcoxon Signed-Rank test between recumbent and standing symptoms

Exercise may aggravate OI in POTS subjects [5–8]. If so, then exercise may induce or aggravate Dizziness and Lightheadedness in CFS. We proposed that exercise would worsen OI symptoms in CFS as a component of PEM, and that POTS CFS would have the worst symptoms. The incremental increases in Recumbent, Standing, and ΔSymptoms for Dizziness and Lightheadedness for each subject were plotted and visually inspected to measure the increase in symptom severity after exercise (Fig. 2). Area under the curve (AUC) was higher in CFS compared to controls for Recumbent, Standing, and ΔSymptoms for Dizziness and Lightheadedness (*p *< 0.05, Mann–Whitney U test). In contrast, OI complaints were equivalent between POTS CFS and CFS without postural tachycardia. Overall, exercise caused an increase in OI symptoms in 22/39 CFS subjects.

The slopes from the individual time courses for recumbent, standing, and ΔSymptoms for Dizziness and Lightheadedness were flat for controls, POTS CFS, and other CFS after exercise. However, time courses for CFS showed more variability in scores over time versus controls. The Root Mean Square Error (RMSE) of the residuals for the post-exercise period was calculated to quantify this variability. RMSE was significantly different between controls and CFS (*p *< 0.01, Mann–Whitney U test) indicating lability of symptoms in CFS and no alteration over time in controls. The variability in symptoms after exercise supports OI as a component of post-exertional malaise.

### Does exercise induce OI symptoms and postural tachycardia (START) in CFS and controls?

Because Dizziness and Lightheadedness were present in POTS and other CFS subjects, we proposed that the START subjects who developed transient postural tachycardia only after exercise may account for the OI symptoms in CFS. START was identified in 28% (7/25) controls and 26% (10/39) CFS subjects (FET, ns).

Symptoms and the incremental changes (ΔSymptoms) after standing up were compared between POTS, STOPP, and START subgroups of CFS and sedentary control subjects. Recumbent symptoms were present in more CFS than control subjects, but the Gracely Box Scores were not significantly different between CFS and controls. Only CFS STOPP had significantly higher Dizziness symptoms while standing than HC STOPP (*p *< 0.05, Tukey’s HSD) (Fig. 2a, c). All CFS groups had significant elevation of Dizziness and Lightheadedness symptoms after standing (*p *< 0.05, Wilcoxon Signed-Rank test).

The individual time courses were visually inspected and showed equivalent trends between CFS POTS, STOPP, and START. Linear regression, AUC, and RMSE showed no significant differences between CFS subgroups. CFS POTS, STOPP, and START subgroups were not significantly different, which indicated that the cardiovascular changes in POTS and START were not associated with worsening OI symptoms. Coincidentally, only the CFS STOPP group, who did have postural tachycardia like the POTS or START groups had significantly elevated symptoms compared to controls. These findings could be attributed to the larger sample size in the STOPP group, the large variance in scores in each group, and the number of CFS subjects who reported no symptoms (6/39). Postural increases in Dizziness and Lightheadedness were a general characteristic of CFS (Fig. 2).

### Can the symptomatic and asymptomatic OI subjects be distinguished?

Receiver operating characteristics (ROC) were examined to identify thresholds that defined significant Dizziness and Lightheadedness complaints. Thresholds were initially set at 1 for Dizziness and Lightheadedness while Recumbent and Standing, but these were heavily weighted by the number of controls with zero complaints. Specificities for recumbent Dizziness and Lightheadedness were 0.98 and 0.96, respectively, and sensitivities were 0.32 and 0.42. Standing Dizziness and Lightheadedness specificities were 0.91 and 0.92, and sensitivities were 0.58 and 0.66, respectively. ΔSymptoms measured the dynamic change in symptoms with posture and had ROC thresholds at 2 for Dizziness and Lightheadedness with specificities of 0.96 and 0.90, respectively, with sensitivities of 0.43 and 0.46, respectively. Thresholds were set at 2 for all variables.

These thresholds stratified control and CFS subjects into three groups: (i) No OI group with no significant (< 2) Dizziness and Lightheadedness complaints while recumbent or standing, (ii) Postural OI group with no complaints while recumbent, but significant complaints after standing, and (iii) Persistent OI group with significant complaints while recumbent and after standing. In controls, 21/25 met No OI criteria and 4/25 met Postural OI criteria. These 4 subjects were identified as CIF subjects. In CFS, 11/39 met No OI criteria, 12/39 met Postural OI criteria, and 16/39 met Persistent OI criteria.

**Fig. 3:**
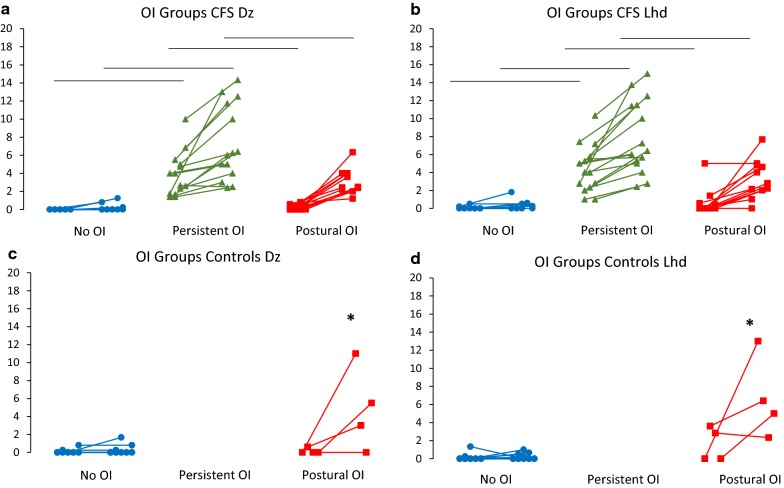
Recumbent and Standing Dizziness and Lightheaded Symptoms in Stratified Groups. Controls and CFS were stratified based on ROC thresholds of 2. Four controls had Dizziness and Lightheadedness symptoms only when standing. The other 21 controls were asymptomatic. Eleven CFS subjects reported scores < 2 while recumbent and standing; 8 had no Dizziness and 6 had no Lightheadedness. For Persistent OI, 16 reported recumbent and standing symptoms ≥ 2, and, in Postural OI, 12 reported only standing complaints ≥ 2. CFS subjects with recumbent and standing complaints ≥ 2 had significantly higher symptoms compared CFS subjects with no symptoms and those only with standing symptoms. Lines indicate trend of score from recumbent to standing. *p < 0.05 Mann–Whitney U test Postural OI controls vs. No OI controls. Black bars indicate *p *< 0.05, Mann–Whitney U test vs. CFS No OI and CFS Persistent OI CFS group for recumbent and standing symptoms

Before and after exercise, the CFS Persistent OI subjects had significantly higher Recumbent and Standing Dizziness and Lightheadedness than the No OI and Postural OI groups (*p *< 0.0001, Mann–Whitney U test). The CFS Postural OI group had significantly higher Dizziness and Lightheadedness than the CFS No OI group only after standing for 5 min (*p *< 0.0001, Mann–Whitney U test). The CFS Persistent OI and Postural OI groups were equivalent for ΔSymptoms and were both significantly higher than the CFS No OI group (*p *< 0.0001, Mann–Whitney U test) (Fig. 3).

### Did exercise affect OI symptoms?

The effects of exercise on Recumbent, Standing, and ΔSymptoms were assessed by AUC, RMSE, and linear regression of individual time courses. Recumbent symptoms were made worse after exercise in the Persistent OI compared to other CFS groups based on AUC (*p *< 0.005, Mann–Whitney U test). The lability of symptoms was also significantly worse in Persistent OI (RMSE, *p *< 0.005, Mann–Whitney U test). Standing and ΔSymptoms Dizziness and Lightheadedness were equivalent between Persistent OI and Postural OI groups. Both had significantly higher AUC and RMSE than No OI for these variables (*p *< 0.0001, Mann–Whitney U test). In contrast, controls did not have labile symptoms by RMSE and only the 4 Postural OI subjects had significantly higher AUC than the other controls.

### Were there demographic differences between OI groups?

There were no significant differences in demographics between these groups (Table 2). No significant differences were observed between OI groups in CFS in questionnaires assessing autonomic dysfunction, fatigue, quality of life or anxiety. Significant differences were seen between controls and CFS subjects for cognition, fatigue, pain, and quality of life (*p *< 0.05, Tukey’s HSD). The CFS Persistent OI group had significantly higher scores on the COMPASS-31 than the CFS No OI group and scored significantly higher on headache, neurological, and ear/sinus-related questions on a systemic complaints questionnaire (*p *< 0.05, Tukey’s HSD). These results suggest that autonomic dysfunction, neurological issues, and vestibular mechanisms are related to elevated orthostatic symptoms.

**Table 2:** OI Group Demographic

	No OI Controls N = 21	Postural OI Controls N = 4	No OI CFS N = 11	Persistent OI CFS N = 16	Postural OI CFS N = 12	ANOVA
Age	44 ± 18	41 ± 25	44 ± 9	51 ± 15	46 ± 13	ns
Males	13	1	3	5	3	ns
Females	8	3	8	11	9	ns
BMI	28.74 ± 4.56	22.15 ± 1.71	25.8 ± 4.7	25.9 ± 4.6	27.6 ± 7.2	ns
COMPASS-31 [13]						
COMPASS-31 Sum	12.06 ± 8.60*	29.09 ± 7.96	22.40 ± 15.26	36.45 ± 9.10	27.25 ± 15.37	ns
OI	6.11 ± 7.10*	19.00 ± 11.49	12.40 ± 10.23	17.14 ± 8.22	13.45 ± 8.81	ns
Vasomotor	0*	0	0	0.65 ± 0.93	0.98 ± 0.97	*p *< *0.001*
Secretomotor	1.47 ± 2.03*	2.68 ± 3.21	1.50 ± 2.68	5.66 ± 3.72	2.73 ± 3.72	*p *< *0.001*
GI	3.01 ± 3.52*	5.80 ± 3.30	5.09 ± 3.62	9.37 ± 3.48	6.90 ± 4.89	*p *< *0.001*
Bladder	0.58 ± 1.19	0.28 ± 0.56	0.78 ± 1.18	1.11 ± 1.63	1.21 ± 1.44	ns
Pupillomotor	0.89 ± 0.57*	1.33 ± 0.90	2.63 ± 1.17	2.50 ± 0.61	1.97 ± 0.71	*p *< *0.001*
CFS_Q_Fatigue [20]	0.94 ± 1.11*	2.50 ± 1.29*	3.27 ± 1.19	3.53 ± 0.64	3.42 ± 0.67	*p *< *0.001*
CFS_Q_Sum8 [20]	4.72 ± 5.17*	11.25 ± 2.75*	16.64 ± 6.30	19.60 ± 5.05	17.17 ± 4.45	*p *< *0.001*
Chalder_11 [27]	11.63 ± 4.13*	18.00 ± 7.66*	20.36 ± 7.10	23.73 ± 5.79	24.00 ± 6.34	*p *< *0.001*
SF-36 [28]						
Physical functioning	88.68 ± 21.72*	87.50 ± 10.41*	52.73 ± 33.57	44.33 ± 24.12	42.50 ± 22.21	*p *< *0.001*
Role—physical	90.79 ± 12.54*	25.00 ± 48.99*	20.45 ± 40.03	6.67 ± 17.59	2.08 ± 7.22	*p *< *0.001*
Role—emotional	92.28 ± 21.02	50.00 ± 57.54	57.58 ± 47.35	68.89 ± 46.23	83.33 ± 38.92	ns
Vitality	75.79 ± 19.51*	36.75 ± 20.55*	40.73 ± 27.46	28.07 ± 23.85	37.00 ± 18.12	*p *< *0.001*
Mental Health	77.26 ± 13.54	57.00 ± 27.59	63.64 ± 10.95	66.93 ± 20.03	72.00 ± 17.14	ns
Social function	88.16 ± 17.42*	46.88 ± 27.72*	43.18 ± 33.24	27.50 ± 21.75	29.17 ± 26.29	*p *< *0.001*
Bodily pain	87.63 ± 15.79*	76.50 ± 37.11*	48.82 ± 30.35	42.40 ± 22.70	50.17 ± 29.29	*p *< *0.001*
General Health	75.79 ± 19.51*	36.75 ± 20.55*	40.73 ± 27.46	28.07 ± 23.85	37.00 ± 18.12	*p *< *0.001*

There were no significant differences in demographics between groups. The 3 CFS groups were significantly different from both SC groups for measures of autonomic dysfunction, cognitive dysfunction, fatigue, pain, and quality of life (*p *< 0.05, Tukey’s HSD). The CFS Persistent OI group had significantly higher scores on the COMPASS-31 than the CFS No OI group and scored significantly higher on headache, neurological, and ear/sinus-related questions on a systemic complaints questionnaire (*p *< 0.05, Tukey’s HSD)

## Discussion

The purpose of this study was to characterize orthostatic intolerance and to determine the effect of exercise on orthostatic intolerance in CFS. Prior literature has established an association between OI symptoms and CFS, and comorbidity of POTS (OI symptoms plus postural tachycardia) and CFS [29–32]. The association was considered strong enough that OI was included as one of two manifestations that must accompany fatigue, post-exertional malaise, and unrefreshing sleep in order to meet Systemic Exertional Intolerance Disease criteria [4].

Most testing for OI in CFS has utilized head-up tilt (HUT) testing rather than active stand protocols. In contrast, the instruments used to assess autonomic dysfunction and orthostatic intolerance, such as the COMPASS-31 and Orthostatic Grading Scale, only focus on the presence of symptoms while standing. They do not address symptoms that are present during recumbency. We found that 41% of CFS had Dizziness and Lightheadedness while recumbent.

One concern with tilt testing is the high rate of false positive test results in controls compared to active stand protocols, which has been reported up to 61% in controls [33–36]. These results suggest that tilt testing may not have sufficient specificity as a provocation to diagnose postural tachycardia. The traditional change in heart rate to diagnose postural tachycardia is an increase ≥ 30 bpm after five minutes of standing. However, ROC analysis of control and POTS subjects is reported to give optimal thresholds of ΔHR = 29 bpm after 10 min of standing and ΔHR = 38 after 10 min of HUT testing. At 30 min, the optimal thresholds were ΔHR = 34 for active standing and ΔHR = 47 for HUT [37]. The higher ΔHR reported during HUT may explain the high false positive rate for postural tachycardia in controls.

The leading hypothesis to explain OI symptoms is cerebral hypoperfusion. However, there were no differences for cerebral blood flow velocities measured by transcranial Doppler between POTS, orthostatic hypotension, OI patients with normal HUT, and controls [38]. An alternative suggested by a “good day–bad day” study was cardiovascular dysfunction with significantly higher heart rates at rest, and significantly lower left ventricular end-diastolic diameter, and stroke index observed on “bad” days compared to “good” days. Impaired cardiovascular responses to standing has also been reported [39, 40].

Our study had several novel observations about OI symptoms in CFS. We found that Dizziness and Lightheadedness were present in 41% (16/39) of CFS subjects while recumbent and 72% (28/39) after standing up for 5 min. We initially proposed that OI symptoms would be higher in CFS subjects with POTS because Dizziness, Lightheadedness, fatigue, and exercise intolerance are components of POTS [5–7]. Symptomatic POTS, defined by postural tachycardia with orthostatic symptoms while standing, was found in 1/25 control and 6/39 CFS subjects. In additional, exercise induced the novel START phenotype with transient, postural tachycardia induced by exercise in 8/25 controls and 10/39 CFS subjects. None developed Neurally Mediated Hypotension. Remarkably, no significant differences in OI symptoms were seen before or after exercise in CFS POTS and START compared to CFS subjects without postural tachycardia (STOPP). These findings lead to the conclusion that postural tachycardia may be a comorbidity, but not the cause, of Dizziness and Lightheadedness symptoms in orthostatic intolerance in CFS.

Instead, based on ROC and thresholds at 2 on the Gracely Box Scale, we defined groups with OI symptoms while recumbent and after standing (Persistent OI, 41%) and those with OI symptoms only after standing up (Postural OI, 31%). Richardson et al. performed a similar stratification of CFS subjects using a weighted standing time. Standing duration, coupled with a self-reported test difficulty score was able to be quantified as a component of OI in this cohort, offering a novel way to evaluate OI in CFS subjects [41]. Exercise exacerbated OI symptoms in both the Persistent and Postural OI groups, with Persistent OI being the most symptomatic. A significant feature of CFS was the lability of symptoms over time, measured by RMSE, compared to the asymptomatic responses of controls. Further work may show that lability in symptoms that is made worse by exertion may distinguish CFS from other conditions in the differential diagnosis, and from controls. The increase in lability of OI symptoms may also be a component of post-exertional malaise, which has been difficult to quantify and lacks a consensus definition [42–44]. The variation in symptoms observed here suggests that observations should be recorded for several hours after provocation, such as exercise or HUT, to quantify this lability. Further work is required to investigate if post-exercise lability is observed in other symptoms in CFS, such as fatigue, pain, and cognition, and if these effects are found in other illnesses like GWI.

## Conclusions

We investigated orthostatic intolerance in chronic fatigue syndrome and introduced the Gracely Box Scale as an easily applied instrument to measure Dizziness and Lightheadedness symptom severity in a clinical setting. Orthostatic symptoms were not explained by POTS in our participants. Exercise induced transient, postural tachycardia in both control and CFS subjects (START), but the occurrence of START did not account for orthostatic symptoms. These findings suggest that postural tachycardia and orthostatic symptoms may be separate co-morbidities of CFS. ROC analysis established thresholds of 2 on the Gracely Box Scale for significant Dizziness and Lightheadedness in CFS. These thresholds stratified our participants into three groups: (i) No OI group with no significant Dizziness and Lightheadedness, (ii) Postural OI with symptoms only after standing, and (iii) Persistent OI with significant recumbent and standing complaints. Persistent OI had greater lability and higher OI complaints than other CFS subjects. Dizziness and Lightheadedness while recumbent is an important finding in chronic fatigue syndrome and should be measured when doing a clinical evaluation to diagnose orthostatic intolerance.

## Data Availability

The datasets used and/or analyzed during the current study are available from the corresponding author on reasonable request.

## Abbreviations

CFS: Chronic fatigue syndrome
SEID: systemic exertion intolerance disease
OI: orthostatic intolerance
IOM: Institute of Medicine
HR: heart rate
POTS: postural orthostatic tachycardia syndrome
START: Stress Test Activated Reversible Tachycardia
STOPP: Stress Test Originated Phantom Perception
GWI: Gulf War illness
HUT: head-up tilt
PEM: post-exertional malaise
